# The China National GeneBank Sequence Archive (CNSA) 2024 update

**DOI:** 10.1093/hr/uhaf036

**Published:** 2025-02-06

**Authors:** Weiwen Wang, Cong Tan, Ling Li, Xia Li, Lei Zhang, Xiaoqiang Li, Jieyu Wang, Ziyi He, Tao Yang, Kailong Ma, Qingjiang Hu, Wenzhen Yang, Zhiyong Li, Mingwen Zhang, Wensi Du, Fan Yang, Zhicheng Xu, Xizheng Ma, Jiawei Tong, Jia Cai, Cong Hua, Fengzhen Chen, Lijin You, Liang Li, Wenjun Zeng, Bo Wang, Xun Xu, Xiaofeng Wei

**Affiliations:** Guangdong Provincial Genomics Data Center, BGI Research, Beishan Road, Yantian District, Shenzhen 518120, China; China National GeneBank, BGI Research, Jinsha Road, Dapeng District, Shenzhen 518120, China; BGI Research, Beishan Road, Yantian District, Shenzhen 518083, China; State Key Laboratory of Agricultural Genomics, BGI Bioverse, Yunhua Road, Yantian District, Shenzhen 518083, China; Data Application Center, BGI Research, Kejisan Road, Donghu District, Wuhan 430074, China; Guangdong Provincial Genomics Data Center, BGI Research, Beishan Road, Yantian District, Shenzhen 518120, China; China National GeneBank, BGI Research, Jinsha Road, Dapeng District, Shenzhen 518120, China; BGI Research, Beishan Road, Yantian District, Shenzhen 518083, China; China National GeneBank, BGI Research, Jinsha Road, Dapeng District, Shenzhen 518120, China; BGI Research, Beishan Road, Yantian District, Shenzhen 518083, China; Data Application Center, BGI Research, Kejisan Road, Donghu District, Wuhan 430074, China; Data Application Center, BGI Research, Kejisan Road, Donghu District, Wuhan 430074, China; China National GeneBank, BGI Research, Jinsha Road, Dapeng District, Shenzhen 518120, China; BGI Research, Beishan Road, Yantian District, Shenzhen 518083, China; Data Application Center, BGI Research, Kejisan Road, Donghu District, Wuhan 430074, China; China National GeneBank, BGI Research, Jinsha Road, Dapeng District, Shenzhen 518120, China; BGI Research, Beishan Road, Yantian District, Shenzhen 518083, China; China National GeneBank, BGI Research, Jinsha Road, Dapeng District, Shenzhen 518120, China; BGI Research, Beishan Road, Yantian District, Shenzhen 518083, China; China National GeneBank, BGI Research, Jinsha Road, Dapeng District, Shenzhen 518120, China; BGI Research, Beishan Road, Yantian District, Shenzhen 518083, China; China National GeneBank, BGI Research, Jinsha Road, Dapeng District, Shenzhen 518120, China; BGI Research, Beishan Road, Yantian District, Shenzhen 518083, China; Data Application Center, BGI Research, Kejisan Road, Donghu District, Wuhan 430074, China; Data Application Center, BGI Research, Kejisan Road, Donghu District, Wuhan 430074, China; China National GeneBank, BGI Research, Jinsha Road, Dapeng District, Shenzhen 518120, China; BGI Research, Beishan Road, Yantian District, Shenzhen 518083, China; China National GeneBank, BGI Research, Jinsha Road, Dapeng District, Shenzhen 518120, China; BGI Research, Beishan Road, Yantian District, Shenzhen 518083, China; China National GeneBank, BGI Research, Jinsha Road, Dapeng District, Shenzhen 518120, China; BGI Research, Beishan Road, Yantian District, Shenzhen 518083, China; China National GeneBank, BGI Research, Jinsha Road, Dapeng District, Shenzhen 518120, China; BGI Research, Beishan Road, Yantian District, Shenzhen 518083, China; Data Application Center, BGI Research, Kejisan Road, Donghu District, Wuhan 430074, China; Data Application Center, BGI Research, Kejisan Road, Donghu District, Wuhan 430074, China; Data Application Center, BGI Research, Kejisan Road, Donghu District, Wuhan 430074, China; BGI Research, Beishan Road, Yantian District, Shenzhen 518083, China; China National GeneBank, BGI Research, Jinsha Road, Dapeng District, Shenzhen 518120, China; BGI Research, Beishan Road, Yantian District, Shenzhen 518083, China; China National GeneBank, BGI Research, Jinsha Road, Dapeng District, Shenzhen 518120, China; BGI Research, Beishan Road, Yantian District, Shenzhen 518083, China; China National GeneBank, BGI Research, Jinsha Road, Dapeng District, Shenzhen 518120, China; BGI Research, Beishan Road, Yantian District, Shenzhen 518083, China; China National GeneBank, BGI Research, Jinsha Road, Dapeng District, Shenzhen 518120, China; BGI Research, Beishan Road, Yantian District, Shenzhen 518083, China; BGI Research, Beishan Road, Yantian District, Shenzhen 518083, China; Guangdong Provincial Genomics Data Center, BGI Research, Beishan Road, Yantian District, Shenzhen 518120, China; China National GeneBank, BGI Research, Jinsha Road, Dapeng District, Shenzhen 518120, China; BGI Research, Beishan Road, Yantian District, Shenzhen 518083, China

## Abstract

The China National GeneBank Sequence Archive (CNSA) is an open and freely accessible curated data repository built for archiving, sharing, and reutilizing of multiomics data. The remarkable advancement in sequencing technologies has triggered a paradigm shift in life science research. However, it also poses tremendous challenges for the research community in data management and reusability. With the dramatic advance of sequencing technologies like spatial transcriptome sequencing, it brings an unprecedented explosion in sequence data and new requirements for data archiving. CNSA was established in 2017 as one of the fundamental infrastructures to offer multiomics data archiving for the worldwide research community. Here, we present the state-of-the-art enhancements of CNSA encompassing the dramatical increase of varied types of data, the latest features and services implemented in CNSA as well as consistent efforts supporting global cooperation in biodiversity preservation and utilization. CNSA provides public archiving and open-sharing services for sequencing data and relevant metadata including genome, transcriptome, metabolism, and proteome from single-cell (also spatial resolved) level to individual and population level, as well as further analyzed results. As of 2024, CNSA has archived >16.3 petabytes of data and provided the data curation, preservation, and open-share service for >1581 publications from >560 institutions. It plays a pivotal role in supporting global scientific projects such as the 10 000 Plant Genomes Project. So far, CNSA has been recommended by various academic publishers such as Cell, Elsevier, and Oxford University Press. CNSA is accessible at https://db.cngb.org/cnsa/.

## Introduction

The dramatic development of sequencing technology has brought a paradigm shift in academic research in life science. Taking advantage of these advancements, the global scientific community has organized various large-scale scientific initiatives, such as the Earth BioGenome Project (EBP) [1, 2] and the Vertebrate Genomes Project (VGP) [3], enabling a holistic understanding of gene functions, genetic diseases, phylogenetics, and biodiversity by analyzing extensive biological data. These large-scale projects have led to an unprecedented explosion in multiomics data, which poses great challenges to effectively depositing, archiving, sharing, and reusing these data resources for further reuse. Moreover, biological data is complex, and different research directions result in diverse data. For instance, genome assemblies provide insight into the genetic makeup of organisms. Single-cell data, on the other hand, allows researchers to analyze genetic expression at the individual-cell level. Spatial transcriptomics captures gene expression patterns within distinct spatial contexts, shedding light on dynamic biological processes.

To address these data preservation and open-sharing challenges, several data repositories have been built. These include the Sequence Read Archive (SRA) of the National Center for Biotechnology Information (NCBI) [4], the European Nucleotide Archive (ENA) of the European Bioinformatics Institute (EMBL-EBI) [5], the Sequence Read Archive (DRA) of the DNA Data Bank of Japan (DDBJ) [6], and the Genome Sequence Archive (GSA) of the National Genomics Data Center (NGDC) [7]. However, the service provided by the few data repositories falls far short of the challenges faced by the increasingly diverse needs in the whole life research community. More diverse and practical efforts are required to deal with data explosion and the conduction of large-scale genome projects and ensure the security of data resources that benefit all human beings.

**Figure 1:**
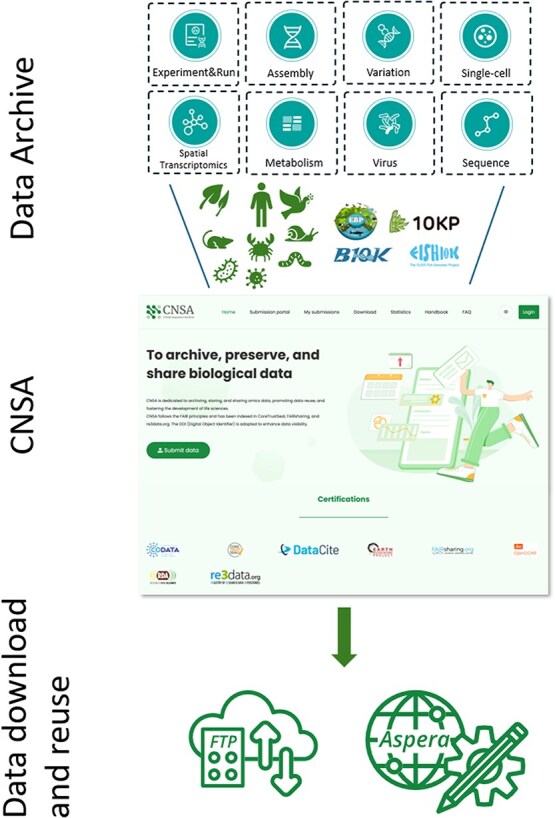
The services of CNSA. CNSA supports the archiving and data sharing of Experiment/Run (fastq data), Assembly, Variation, Single-cell, Spatial Transcriptomics, Metabolism, Virus, and Sequence data across different species ranging from microorganisms to plants, animals, and humans, as well as the data from large initiatives, such as EBP, 10 KB, and B10K.

The China National GeneBank Sequence Archive (CNSA) is a curated data repository built for public archiving and open-sharing services for sequencing data and relevant metadata in life science (Fig. 1). As part of China National GeneBank (CNGB), CNSA was officially put into use and operation in 2017 and first reported in 2020 [8]. CNGB [9] is a global nonprofit fundamental scientific infrastructure and one of the largest centers of DNA sequencing, scientific research, and industry innovation in life science. In recent years, CNSA has engaged with the whole scientific community and played a significant role in supporting global research initiatives, such as the EBP [1, 2], the 10 000 Fish Genomes Project (Fish 10 K) [10], the Bird 10 000 Genomes Project (B10K) [11], and the 10 000 Plant Genomes Project (10KP) [12].

Here, we present the dramatic increase of varied types of data, the latest features and services implemented in CNSA, and its efforts supporting global cooperation in biodiversity preservation and utilization. We also discuss the challenges arising from the rapid data increase faced by CNSA. In short, CNSA will greatly benefit the research community by promoting efficient dealing with and making full use of the exponentially accumulating volume of multiomics data together with the whole research community. All resources are available at https://db.cngb.org/cnsa/.

## Results

### Overview of CNSA

CNSA provides public archiving and open-sharing services for sequence data and relevant metadata of all kinds including genome, transcriptome, metabolism, and proteome from single-cell (also spatial resolved) level to individual and population level, as well as further analyzed results such as assembly, alignments, variation, and gene expression matrices (Fig. 1). To facilitate both Chinese and internationa users, CNSA offers a bilingual data archive system, including English and Chinese, for enhanced accessibility.

**Figure 2:**
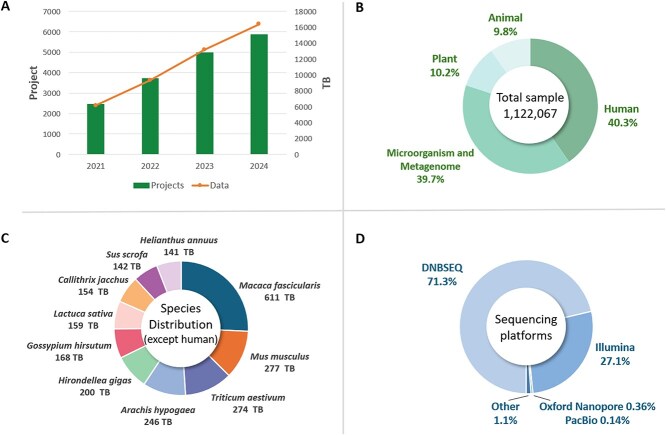
Data statistics of CNSA. A. The growth in project submissions and the annual increase in data volume at CNSA between 2021 and 2024. B. The classification of samples that are submitted to CNSA. C. Top 10 species by data volume (excluding *Homo sapiens*) in CNSA. D. Distribution of data volumes across sequencing platforms in CNSA. This distribution is calculated based on sample sequencing.

As of August 2024, CNSA received 5836 project submissions from 564 institutions, totaling 16.3 petabytes (PB) of data, including 1 122 067 samples, 1 766 269 sequencing data across 47 distinct sequencing platforms, and 125 855 assemblies (Fig. 2). These data encompass information from 7521 species, including 4499 plants, 1520 animals, and 1526 microorganisms. The data can be downloaded through standard methods (FTP and HTTPS) as well as the high-speed Aspera transfer protocol, which could be up to 100× faster than FTP. Notably, CNSA supports data archiving and publication for 1581 manuscripts across 380 journals, including Nature, Cell, and Science. Additionally, CNSA has received ~15 million website visits, engaging users from 181 countries and regions in the past 7 years.

### New feature of CNSA

Since its initial report in 2020, tremendous improvements in several aspects have been made in CNSA to meet the various requirements of the research community. Firstly, through a series of system upgrades, CNSA has enhanced user experience and international recognition, attracting an increasing number of users. The archived data volume has increased from ~2 PB in 2020 to 16.3 PB currently, showing a rapid growth rate exceeding 3 PB per year (Fig. 2A). For instance, in 2023, up to 3.8 PB of sequencing data generated from 1249 projects was submitted and archived in CNSA.

Regarding data types, except for Experiment/Run, Assembly, and Variation data types, CNSA now also supports additional data types, including Metabolism, Single-cell, Virus, Sequence, and Spatial Transcriptomics data (Table 1). Compared to other data types, spatial transcriptomics data is unique and complex. It includes not only normal sequencing reads but also barcodes, histological images, and other information. To enhance data reusability, CNSA has developed a spatial transcriptomic data archiving system to archive all information relevant to spatial transcriptomic sequencing data and results (Supplementary Fig. S1). This archiving system is specifically designed for spatial transcriptomic data, aiming to maximize the display of spatial transcriptomic data information and facilitate data reuse. This includes the Project, Sample, Tissue section, Experiment & Run, and Analysis results. Tissue section is a new and unique object exclusive to spatial transcriptomic technology, which carries specific information content, such as tissue section size, section thickness, slice position, and cryosectioning temperature. Besides, more detailed information and instruction for the data structures that could be submitted into CNSA archive system in the submission page (https://db.cngb.org/stomics/submission). All spatial transcriptomics submitted to CNSA can be linked to our visualization system to generate a freely accessible visualized dataset [13] Specifically, we annotated cell types, identified spatial regions and genes, and performed cell–cell interaction analysis for these datasets. Researchers can explore the analysis results and visualize the expression data through the dataset module. As of August 2024, the spatial transcriptomic data archiving system has archived 59 projects, amounting to 65 TB of data.

**Table 1:** Overview of CNSA archived data.

**Data type**	**Description**	**Supported format**	**Records**
Experiment&Run	The raw sequencing data, including the description of sample-specific sequencing library, instrument, and sequencing methods.	FASTQ, BAM, CRAM, SFF, PacBio_HFD5, etc	1 766 269
Assembly	The assembled genomic sequence and its annotation files.	FASTA, and annotation (such as GFF, GTF)	125 855
Variation	Genomic variations from any species, including single nucleotide polymorphisms, short insertions/deletions, and genomic structural variations, etc.	VCF	121 703
Single cell	The analysis results of data generated using single-cell technology, including gene expression files, metadata files, cluster files, and other files.	TSV, MM coordinate matrix (such as MTX)	4293
Spatial transcriptomics	The analysis results of data generated using spatial transcriptomics technology, including expression files, annotation files, and image files.	Expression files (such as GEM, GEF), annotation files (such as TSV), image files (such as JPG, PNG, TIFF)	3427
Metabolism	The data for metabolomics.	RAW, ZIP, JDX, CDF, WIFF, JCAMP	11 112
Virus	Viral sequence data	FASTA	3705
Sequence	The sequence of single-gene, mRNA, ncRNA, or synthetic constructs, etc.	GenBank Flat File	20 102

Furthermore, CNSA is dedicated to ensuring data accessibility and reliability and has been acknowledged through certifications from various international organizations since 2020. By following international repository management and open access standards, CNSA has been indexed in FAIRsharing [14], OpenDOAR [15], and re3data [16] as a certified data repository. Until August 2024, CNSA has been recommended by various academic publishers such as Cell, Elsevier, and Oxford University Press and acknowledged by >380 journals. Additionally, as a member of both the World Data System (https://worlddatasystem.org/) and the Research Data Alliance (https://www.rd-alliance.org/), all the process of data curation, archiving, preservation in CNSA is subject to the global standards and requirements. Moreover, CoreTrustSeal [17] certified CNSA as a core trustworthy data repository, affirming CNSA’s ability in data preservation and curation.

### Example of project support

As an internationally recognized large-scale data repository, CNSA has played a pivotal role in the scientific advancement of numerous major initiatives on plants, animals, and microorganisms, facilitating big-data sharing (Table 2). For instance, CNSA is listed as one of the recommended data repositories for the large-scale EBP [1, 2], facilitating the sharing of genomes across various species and laying a new foundation for biological diversity preservation. Additionally, CNSA is an active participant of the SpatioTemporal Omics Consortium (STOC, https://sto-consortium.org/index.html#anchor3), which is a global cooperation consortium for spatial transcriptomics and aims to provide spatiotemporally resolved cellular atlases of all lives. To support the preservation and reuse of spatial transcriptomic data, CNSA has developed spatial transcriptomic data archive standards and a visualization system [13].

**Table 2:** Examples of CNSA-supported initiatives and projects

**Initiatives/Projects**	**Description**	**Related link**
The Earth BioGenome Project	The Earth BioGenome Project is a global initiative that aims to create annotated chromosome-scale reference assemblies for all eukaryotic in the earth.	https://www.earthbiogenome.org/
The 10 000 Fish Genomes Project	The Fish10K (The 10 000 Fish Genomes Project) aimed to sample, sequence, assemble, and analyze 10 000 representative fish genomes under a systematic context within 10 years.	http://fish10k.genomics.cn/
The Bird 10 000 Genomes Project	The Bird 10 000 Genomes (B10K) Project plans to generate representative draft genome sequences from all extant bird species. The B10K project will complete a genomic-level tree of the entire bird species, decode the relationship between genetic variation and phenotypic variation, evaluate the impact of various ecological factors and human influence on species evolution, and unveil the demographic history.	https://b10k.genomics.cn/
The 10 000 Plant Genomes Project	The 10 000 plant Genomes Project(10KP) aims to sequence >10 000 genomes representing every major clade of plants and eukaryotic microbes. This project aims to address fundamental questions about plant evolution.	https://db.cngb.org/10kp/
The 10 000 Medicinal Plant Project	The 10 000 Medicinal Plants Project is designed based on the data of Guangxi Innovation-Driven Development Special Project and Construction of Big Data Centre of Medicinal Plants. The project aims at building the largest big-data center of medicinal plants in the world, interpreting the relationship between the big data of medicinal plants and the pharmaceutical efficacy.	https://db.cngb.org/10kmp/
The *Arabidopsis Thaliana* Database	The Arabidopsis Genome Database encompasses gene annotations, genetic variations, gene expression data, and microbiome information of *Arabidopsis*, covering >10 million variant loci across 1135 samples.	https://db.cngb.org/genomics/arabidopsis/
The Cycas Genome Project	The Cycad Genome Project is an integration of genomic data from cycads and other related seed plants, including raw sequencing data, assembly, and annotation, encompassing information from 339 cycad species.	https://db.cngb.org/codeplot/datasets/public_dataset?id=PwRftGHfPs5qG3gE
The Lettuce Project	The Lettuce Project created the LetuceDB database for cultivated lettuce containing 445 *Lactuca* accessions. The database integrated the multidimensional data into six modules: germplasm, genome, variome, phenome, microbiome, and spatial–temporal transcriptome.	https://db.cngb.org/lettuce/

In terms of plant sciences, CNSA has provided critical support for large-scale initiatives such as the 10 000 Medicinal Plant (10KMP) project and the10KP [12]. The 10KMP aims to sequence >10 000 genomes of medicinal plants, which could promote innovation in medicinal plant resources and accelerate the development of traditional Chinese medicine. To date, ~2672 plant species have been sequenced, yielding ~19.56 TB of data (https://db.cngb.org/10kmp/). CNSA has worked closely with the 10KMP committee from the project initiative, designed data storage, and managed and further open share of these invaluable data resources. Another significant undertaking is the 10KP project, launched in 2018 with the objective of sequencing and analyzing major plant genomes from every major clade [12]. The 10KP project is currently in progress and has already sequenced >2500 plant species, covering ~90% of the bryophyte family level and ~70% of the angiosperm order level. Numerous research outputs have been published, and their data are publicly available on CNSA [18–26].

In addition to these large-scale initiatives, CNSA plays a pivotal role in supporting research on both model plants and horticultural plants. Specifically, for the model organism *Arabidopsis thaliana*, CNSA has collaborated with researchers to construct the *A. thaliana* database, providing comprehensive biological information about *A. thaliana* based on the data they deposited on CNSA (https://db.cngb.org/genomics/arabidopsis/) [27].

Horticultural plants are integral to human diets and landscapes. However, their cultivation faces numerous challenges such as pests, diseases, environmental stressors, and consumer demands for higher quality and safety standards. To address these issues, CNSA supports a wide range of horticultural projects that facilitate data sharing and reusability, enhancing research and breeding efforts. For instance, Cycads occupy a distinguished position in horticulture as ancient and ornamental plants with significant historical and esthetic value. A large Cycads project, which analyzed 339 Cycads species, has deposited and shared its dataset on CNSA [28]. This comprehensive dataset not only supports conservation efforts but also provides invaluable resources for researchers studying the genetic diversity, evolutionary history, and adaptive traits of Cycads.

**Figure 3:**
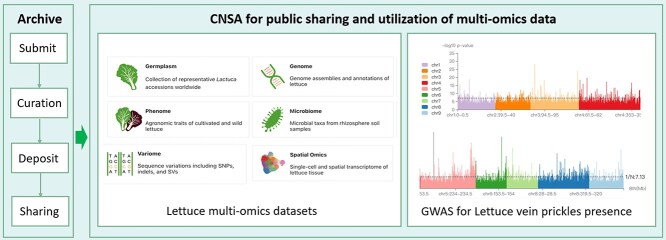
An example of CNSA’s data sharing and reuse for horticultural plants. CNSA plays a pivotal role in archiving, curating, and visualizing diverse data types to enhance the openness and accessibility of horticultural plant data. For instance, CNSA has comprehensively archived multiomics datasets for lettuce, encompassing germplasm information, genome assemblies, phenotypic data, microbiome profiles, variome data, and spatial omics. To maximize the utility of these datasets, CNSA has developed interactive visualizations, including a GWAS that identifies genetic variants associated with the presence of lettuce vein prickles.

Due to the complexity and numerous traits that require investigation in horticultural plants, multiomics data analysis, which encompasses genomics, transcriptomics, proteomics, metabolomics, and other omics, is useful for providing a comprehensive view of the genetic architecture underlying complex traits such as yield, flavor, texture, color, and disease resistance. CNSA provides multiomics data archive, such as raw sequencing data, genome assembly data, variation data, metabolic data, and spatial transcriptomic data, to support advanced studies in horticultural plant science. For instance, a multiomics project focused on lettuce exemplifies the power of this integrated approach. This project has generated multiomics data from 445 cultivated varieties, uncovering key insights into the domestication history and genetic diversity of lettuce [29]. The comprehensive dataset, archived on the CNSA, includes genomic sequences, phenotypic information, variation information, and spatial omics. To further enhance the utility of these data, CNSA, in collaboration with data submitters, has developed a specialized database, LettuceDB (https://db.cngb.org/lettuce/) [30], which offers advanced visualization tools and user-friendly interfaces for exploring and analyzing the multiomics data (Fig. 3). One notable application of this resource is the integration of genome-wide association studies (GWAS) with expression profiling. By combining GWAS data with detailed expression profiles, researchers can identify candidate genes associated with specific phenotypes, such as disease resistance, yield, and nutritional content. This integrative approach enables the precise mapping of genetic variants that influence important agricultural traits, facilitating the discovery of novel genes and pathways. This lettuce resource supports research into lettuce’s genetic architecture and facilitates the identification of valuable traits for breeding programs. In sum, CNSA promotes the openness and accessibility of horticultural plant data, supporting related research and fostering innovation in the field of horticultural plant science.

### FAIRness of CNSA

FAIR principles emphasize the findability, accessibility, interoperability, and reusability of data, providing researchers with a framework for effectively managing and sharing data. As one of the global biological data repositories, CNSA actively implements FAIR principles to ensure that its data meets these principles. To enhance the findability of data/metadata, CNSA follows the DataCite Metadata Schema to make the Digital Object Identifier (DOI) for each public project, and each metadata entry, such as sample or assembly, represented by a unique and permanent accession ID assigned by CNSA itself. The accession IDs are indexed and searchable in CNSA, providing access to metadata and data files. Moreover, CNSA offers reviewer links to enable editors and reviewers to access unpublished projects for manuscript review.

To enhance the reusability of data, quality checks are conducted on both the metadata and data files, and the MD5 value of the data file is provided for verifying the integrity of downloaded files. Data files can be downloaded through multiple ways, such as FTP, Aspera, and HTTPS. All public data of CNSA are hosted on an FTP server and represented in machine-accessible formats. The public data from CNSA are freely available for use, but proper citation is required.

CNSA adheres to universally acceptable controlled vocabularies and standards to ensure the interoperability of data. CNSA employs the Minimum Information about Any (x) Sequence (MIxS) standards, developed by the Genomic Standards Consortium (GSC) [31], to detail nucleic acid sequence origins, environmental context, and processing procedures. This framework, already captured by the International Nucleotide Sequence Database Collaboration (INSDC) [32], encompasses three core standards established by the GSC: Minimum Information about a Genome Sequence (MIGS) [33], Minimum Information about a Metagenomic Sequence (MIMS) [33], and Minimum Information about a Marker Gene Sequence (MIMARKS) [34]. MIGS captures essential contextual information about the experimental method and spatiotemporal context, such as latitude, longitude, and time for sample collection, while MIMS expands the minimum information to include metagenome measurements, such as sampling temperature and pH. MIMARKS further broadens MIGS/MIMS with experimental contextual data such as PCR primers and conditions, and target gene names. In summary, CNSA’s commitment to these controlled vocabularies and standards ensures the understandability of archived data, facilitating the findability, accessibility, interoperability, and reusability of data.

### Data/metadata submission and curation

**Figure 4:**
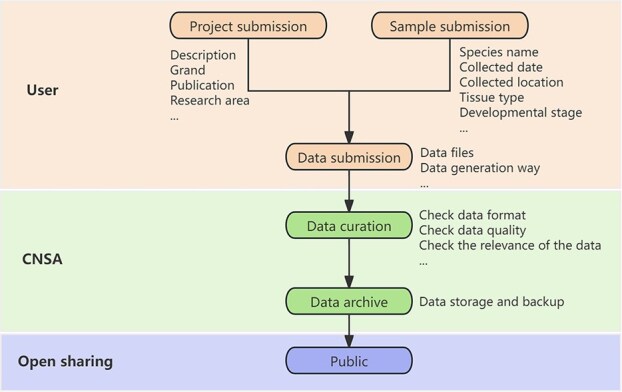
The entire data submission process. Users first need to complete the project and sample submission, including the project description, grant information, sample species name, sample collected location, etc. Then users need to upload the data file along with the corresponding metadata. Once CNSA receives the data, CNSA curators will perform the data quality and format check. Next, the data will be archived and with regular backup. The project and its associated sample/data will be made public based on the release date set by the users.

To complete the data submission process, users are required to submit project information first, including the project title and description, followed by the completion of sample submission (Fig. 4). CNSA mandates that users furnish sufficient information such as species name and sample collection location to guarantee data preservation and reusability. Subsequently, users can submit the data to establish the connection between the project and the sample, thus finalizing the entire data submission process. Currently, CNSA supports eight data types ranging from raw sequencing data to secondary analysis outcomes, encompassing Experiment/Run, Assembly, Variation, Metabolism, Single cell, Virus, Sequence, and Spatial transcriptomics. In terms of data formats, CNSA generally accepts submissions in widely recognized formats like FASTQ, FASTA, VCF, and gene expression matrices. These formats are universally accessible across various operating systems and typically do not necessitate specialized software for viewing, ensuring the interoperability and reusability of data. For supplementary data such as code or image files, CNSA provides an offline submission option, wherein users need to contact the CNSA curator for archiving, promoting data integrity and user-friendly interaction.

Each submission undergoes automated quality control processes and manual review to ensure relevance and clarity. In general, each submission will be processed in 1–3 working days. CNSA will contact users if there are any issues identified in the submissions. Upon successful archiving, the project and its associated sample/data will be made public based on the release date set by the users.

## Discussion

CNSA is dedicated to establishing a comprehensive biological multiomics data repository for promoting data archiving, accessibility, and reusability. Currently, CNSA has curated an extensive archive of 16.3 PB of data encompassing thousands of species from >560 institutions, supporting the publication of 1581 papers across 380 journals. By adhering to international standards such as the FAIR principles and GSC standards, CNSA upholds high levels of data submission, curation, and sharing. Moreover, CNSA’s active involvement in global initiatives like the EBP, B10K, 10KMP, and 10KP enhanced its pivotal role in advancing big-data sharing in life science. In short, CNSA is committed to promoting curation, archiving, preservation, and sharing of multiomics data and advancing the rapid development of research in the life sciences.

The rise of extensive initiatives and rapid progress in sequencing technologies has led to a substantial increase in data volumes. For example, the 10KP project has already generated ~1 PB of data. As a multiomics data repository, CNSA faces challenges for data curation and sharing. To address these challenges, CNSA is preparing to integrate Artificial Intelligence (AI), Application Programming Interface (API), and computational cloud platforms in the near future.

Currently, CNSA has curated >3 PB of data and processed thousands of user inquiries annually, with a steady increase each year. In response to the increase in data archives and user inquiries, CNSA is considering applying AI to improve its processing speed. For the curation, the AI-assisted curation not only ensures better consistency and efficiency of data curation but also facilitates the management of a larger volume of data. Specifically, AI can automatically initiate quality control procedures upon data submission. These procedures conduct quality checks to ensure the integrity and accuracy of the submitted data. If AI identifies issues during the curation, it will automatically send email notifications to users, letting them make necessary corrections based on the errors. This automated error detection and notification mechanism not only reduces the workload of manual review and possible human errors, but also significantly shortens the processing cycle, enhancing the overall efficiency and responsiveness of the data repository. In terms of customer service, AI-assisted customer service will play a role in offering immediate feedback to user inquiries through advanced natural language processing capabilities. This will be achieved through the deployment of virtual customer assistants or chatbots that can understand and respond to requests in real time, providing 24/7 automated support and notably reducing user waiting times, in stark contrast to the current processing time frame of 1–3 working days. This approach will not only improve the user experience but also lead to higher satisfaction rates. Beyond handling general inquiries, AI can interact with the CNSA system, aiding users in addressing issues that necessitate manual intervention in AI’s absence, such as checking submission statuses or obtaining permission to modify or delete specific information. By implementing these AI-driven enhancements, CNSA aims to achieve a more efficient and scalable system, capable of handling the increasing demands of data archives and user inquiries with improved processing speed and user satisfaction.

To promote data accessibility and reusability, CNSA plans to develop APIs that will allow users to programmatically access CNSA data. These APIs will empower users to efficiently retrieve and download specific information from the massive amount of CNSA records and data files. Other biological data repositories such as NCBI and EMBL-ENA have already implemented APIs for seamless programmatic search and retrieval. For example, NCBI’s E-utilities provide extensive access to all Entrez databases for the bulk retrieval of publications, genes, and other NCBI data. Additionally, EMBL-ENA provides programmable access via its REST APIs, enabling users to retrieve samples and other relevant records programmatically. The implementation of APIs at CNSA will not only improve data accessibility but also foster bulk data retrieval processes, promoting efficiency and usability for researchers. Additionally, the APIs could also facilitate the bulk data submission process, particularly for large-scale projects that may involve thousands of samples. Currently, such submissions require users to manually input metadata for each sample, which can be time-consuming and prone to errors. With the implementation of APIs, users can automate the submission of metadata through command lines, streamlining the process. This feature not only enhances the efficiency of data upload but also reduces the potential for human error during data submission.

Furthermore, CNSA plans to develop a comprehensive cloud computational platform including multiomics data archiving, data computing, and reutilizing for the whole research community. By using computational platforms, users can directly access large-scale data without the need for downloading, thus saving local storage costs. Moreover, the robust computational capabilities of the computational platform enable users to conduct analyses directly on CNSA, taking advantage of high-performance computing resources that may not be available locally. This platform will integrate a secure and scalable cloud infrastructure, such as Amazon Web Services (AWS) or Alibaba Cloud, to host its data. This integration will involve setting up virtualized servers that can dynamically allocate computing resources, such as CPU and memory, based on user demand, ensuring high availability and reliability of the service. Additionally, if users require downloads, they can access the data through the cloud computational platform, which could offer greater bandwidth capacity compared to CNSA. This enables users to experience faster download speeds, and effectively reduces the strain on CNSA’s bandwidth resources. The integration of the cloud computational platform into CNSA’s services represents a pivotal step toward a more efficient, scalable, and user-friendly data ecosystem, reinforcing CNSA’s position as a cutting-edge data repository.

## Materials and methods

### CNSA construction

**Figure 5:**
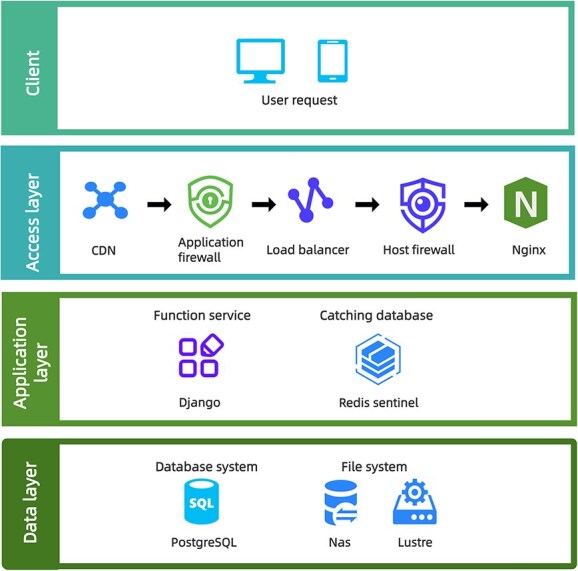
The system architecture of CNSA. The access layer processes user requests through various security measures before reaching the application layer. The application layer is built on Django and utilizes Redis for caching to enhance processing speed. The data layer manages operational data and metadata using a database system and file system, employing PostgreSQL along with NAS and Lustre for efficient data sharing and preservation.

The CNSA system is established in three layers with distinct functions, the access layer, the application layer, and the data layer (Fig. 5). The access layer manages user page access requests and file data access requests. Page access requests are first routed to the nearest CDN (Content Delivery Network) node. For static resource requests, the CDN node can respond immediately; for dynamic resources, the requests pass through the application firewall, load balancer, and individual machine firewall in sequence, ultimately reaching the reverse proxy server (such as Nginx), which then forwards the requests to the application layer for efficient processing. For data access, CNSA offers HTTPS, FTPS, and Aspera services to enhance the efficiency of data download and upload.

CNSA developed the application layer based on Django (https://djangoproject.com). Django is equipped with various Web development components and has strong support from the open-source community, which is a superior platform for Web development. To enhance system performance, CNSA utilized Redis as the caching database to process user sessions and service data. This approach accelerates request processing and reduces the frequency of database access. Additionally, CNSA has deployed a highly available PostgreSQL database cluster to provide a highly reliable data access experience.

To manage the operation data and archived metadata/data, CNSA developed a data layer including a database system and a file system. The database system stores archived metadata and CNSA operation data, such as logs and statistical data. For the file system, CNSA employed the Network-Attached Storage (NAS) and a Distributed Storage System (Lustre), which provide an efficient and reliable solution for data sharing and preservation.

### CNSA security

To ensure the application security, CNSA follows a range of strategies, such as encrypting sensitive information, using ORM to prevent SQL injection, and enforcing strict coding standards to prevent XSS bypass. Every Application undergoes security scans before deployment to ensure the system security. Unauthorized access attempts are blocked by application firewalls (WAF) and host-based firewalls.

Regarding data security, the archived data and public release data are stored separately. To mitigate the risk of data loss, CNSA performs an automatically incremental data backup every day, and conducts regular data recovery tests from backups to validate recovery procedures and ensure the availability of backed-up data. Finally, CNSA employs regular data inspections. If there is any potential data security hazard, an incident assessment and recovery evaluation will be conducted to improve related procedures.

### Data submission and curation

In general, all metadata and data must meet our quality standards. CNSA curators will contact the submitters if their data/metadata submissions have any problems, and the data/metadata submission will not be approved until all issues have been solved.

Regarding metadata quality evaluation, CNSA automatically checks the submitted metadata information with CNSA metadata quality standards. Next, the curator will conduct a manual review to ensure integrity, relevance, and accuracy.

For the data quality assessment, CNSA initiates the process by verifying the MD5 checksum of each uploaded data file to guarantee data integrity. Subsequently, CNSA performs diverse data quality assessments for different data types. For instance, CNSA evaluates genome Assembly data based on criteria like completeness, contamination, and general statistics (such as L50, N50, and GC content), while Experiment&Run data undergo scrutiny for the correctness of the fastq/bam format, average read length, adapter remaining, and other relevant metrics. Finally, CNSA curators manually review the results of these assessments and contact the submitter to address any identified issues.

## Supplementary Material

Web_Material_uhaf036

## Data Availability

All resources are available at https://db.cngb.org/cnsa/.
