# Supplementary figures and images for: The China National GeneBank Sequence Archive (CNSA) 2024 update
Source: Hortic Res. 2025 Feb 6;12(5):uhaf036. doi: 10.1093/hr/uhaf036 (PMC11992327; doi:10.1093/hr/uhaf036)

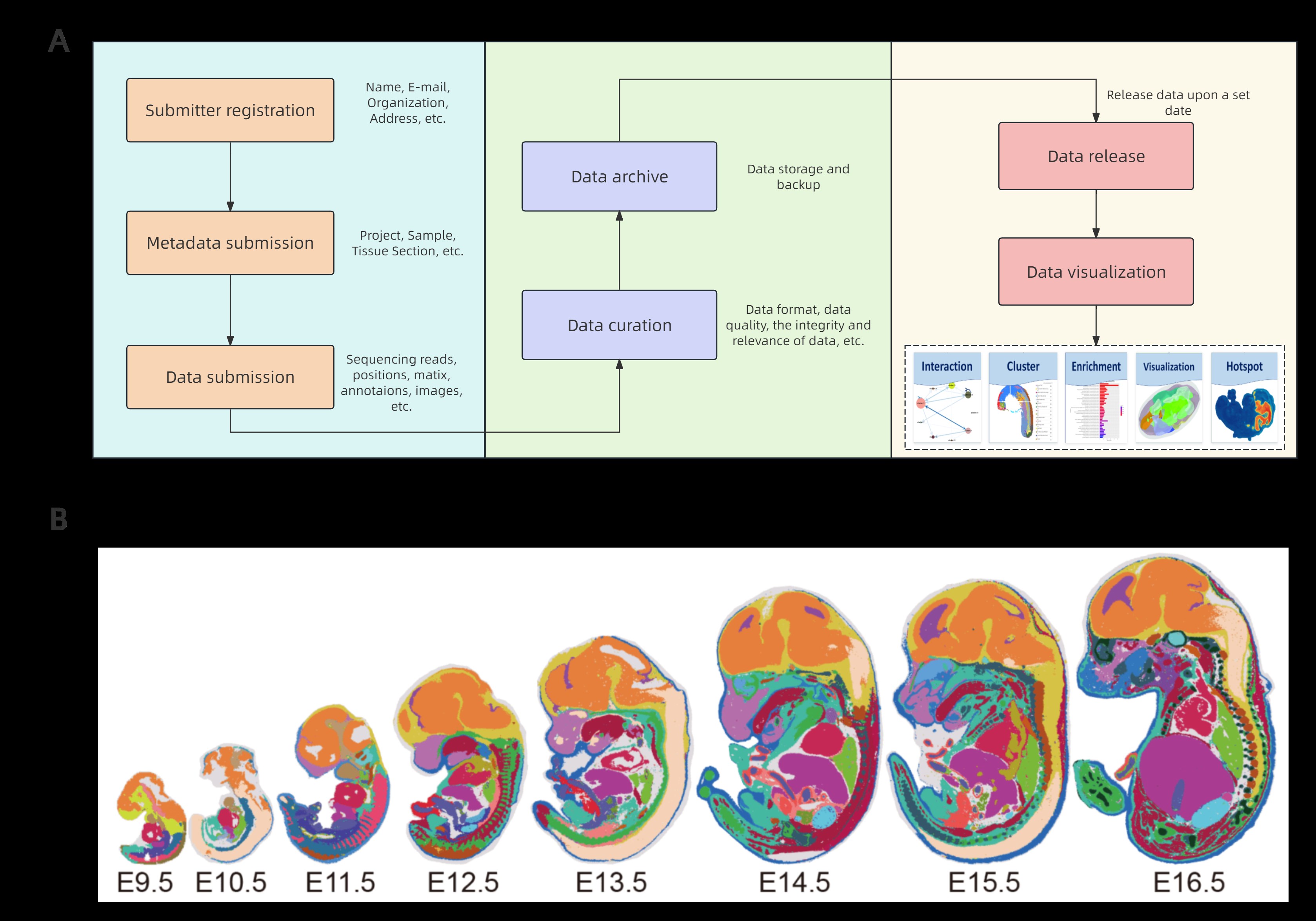

Supplement: Web_Material_uhaf036 [file web_material_uhaf036.jpeg]
